# Family with Sequence Similarity 5, Member C (FAM5C) Increases Leukocyte Adhesion Molecules in Vascular Endothelial Cells: Implication in Vascular Inflammation

**DOI:** 10.1371/journal.pone.0107236

**Published:** 2014-09-24

**Authors:** Junya Sato, Mitsuo Kinugasa, Seimi Satomi-Kobayashi, Kinta Hatakeyama, Aaron J. Knox, Yujiro Asada, Margaret E. Wierman, Ken-ichi Hirata, Yoshiyuki Rikitake

**Affiliations:** 1 Division of Cardiovascular Medicine, Department of Internal Medicine, Kobe University Graduate School of Medicine, Kobe, Hyogo, Japan; 2 Department of Pathology, Faculty of Medicine, University of Miyazaki, Miyazaki, Miyazaki, Japan; 3 Department of Medicine, Physiology, and Biophysics, University of Colorado Denver, Aurora, Colorado, United States of America; 4 Division of Signal Transduction, Department of Biochemistry and Molecular Biology, Kobe University Graduate School of Medicine, Kobe, Hyogo, Japan; Kurume University School of Medicine, Japan

## Abstract

Identification of the regulators of vascular inflammation is important if we are to understand the molecular mechanisms leading to atherosclerosis and consequent ischemic heart disease, including acute myocardial infarction. Gene polymorphisms in *family with sequence similarity 5, member C* (*FAM5C*) are associated with an increased risk of acute myocardial infarction, but little is known about the function of this gene product in blood vessels. Here, we report that the regulation of the expression and function of FAM5C in endothelial cells. We show here that FAM5C is expressed in endothelial cells *in vitro* and *in vivo*. Immunofluorescence microcopy showed localization of FAM5C in the Golgi in cultured human endothelial cells. Immunohistochemistry on serial sections of human coronary artery showed that FAM5C-positive endothelium expressed intercellular adhesion molecule-1 (ICAM-1) or vascular cell adhesion molecule-1 (VCAM-1). In cultured human endothelial cells, the overexpression of FAM5C increased the reactive oxygen species (ROS) production, nuclear factor-κB (NF-κB) activity and the expression of ICAM-1, VCAM-1 and E-selectin mRNAs, resulting in enhanced monocyte adhesion. FAM5C was upregulated in response to inflammatory stimuli, such as TNF-α, in an NF-κB- and JNK-dependent manner. Knockdown of FAM5C by small interfering RNA inhibited the increase in the TNF-α-induced production of ROS, NF-κB activity and expression of these leukocyte adhesion molecule mRNAs, resulting in reduced monocyte adhesion. These results suggest that in endothelial cells, when FAM5C is upregulated in response to inflammatory stimuli, it increases the expression of leukocyte adhesion molecules by increasing ROS production and NF-κB activity.

## Introduction

Ischemic heart disease (IHD) is the leading cause of death worldwide. According to the World Health Organization (http://www.who.int/mediacentre/factsheets/fs317/en/), despite remarkable progress in IHD treatment, more than 7 million people died from IHD in 2008. The central cause of IHD is atherosclerosis of the coronary arteries. Atherosclerosis is considered to be chronic vascular inflammation, initiated by increases in the endothelial expression of leukocyte adhesion molecules, such as E-selectin, intercellular adhesion molecule-1 (ICAM-1) and vascular endothelial adhesion molecule-1 (VCAM-1) [1], [2]. This is often referred to as “endothelial cell activation” and is typically induced by proinflammatory cytokines, such as tumor necrosis factor (TNF)-α and interleukin (IL)-6. It facilitates the recruitment and attachment of circulating leukocytes to the vascular wall. Identification of the regulators of vascular inflammation is important if we are to understand the molecular mechanisms leading to atherosclerosis and consequent IHD.

Family with sequence similarity 5, member C (FAM5C), also known as bone morphogenetic protein (BMP) and retinoic acid (RA)-inducible neural specific protein 3 (BRINP3), was originally identified as a protein homologous to BRINP1 [3]. The FAM5 family of proteins has three members: FAM5A/BRINP1/deleted in bladder cancer protein 1, FAM5B/BRINP2 and FAM5C/BRINP3. These proteins are highly conserved between humans and rodents. The *FAM5C* gene is predominantly and widely expressed in the nervous system, from developmental stages through to adulthood, suggesting a role during the development of the nervous system [3]. However, its expression is not limited to neural tissues; it is also expressed by cultured fibroblasts, vascular smooth muscle cells (SMCs), cancer cells and myoblastic cells [3]–[6]. Kawano et al. reported that ectopically expressed FAM5C localizes to the endoplasmic reticulum in cultured neurons [3]. Shorts-Cary et al. reported that FAM5C localizes in the mitochondria in pituitary gonadotropinomas [4]. However, the mechanism regulating *FAM5C* gene expression remains unknown. In addition, studies to clarify the function of FAM5C have been quite limited. Shorts-Cary et al. showed that its overexpression increased the proliferation, migration, and invasion of pituitary gonadotrope cells [4] and Tanaka et al. showed that FAM5C enhanced the differentiation of cultured osteoblasts [6].

Recently, several studies have reported a link between FAM5C and human diseases. An association between the expression of FAM5C and tongue squamous cell carcinoma [5], the hypermethylation of the *FAM5C* gene in gastric cancer [7], and a genetic association between polymorphisms within the *FAM5C* gene and aggressive periodontitis [8] have been reported. A genome-wide association study identified a genetic association between polymorphisms within the *FAM5C* gene and myocardial infarction, thereby implicating FAM5C as a risk factor for myocardial infarction [9]. An SNP at the 3′ end of the *FAM5C* gene (T allele in rs10920501) correlates with lower incidence of myocardial infarction and lower expression levels of the gene in the aorta [9]. These findings suggest that increased FAM5C levels may be associated with a risk of myocardial infarction. However, little is known about the regulation of FAM5C expression or its function in blood vessels. In cultured human aortic SMCs, expression levels of FAM5C decreased with increasing passage, suggesting a role for this molecule in proliferation and senescence of this cell type [9]. However, the expression and function of FAM5C in other vascular cell types remain unknown. In particular, an association between FAM5C and vascular inflammation has yet to be demonstrated. Therefore, we investigated this issue.

## Materials and Methods

### Antibodies, Plasmids and Reagents

A rabbit anti-FAM5C polyclonal antibody (pAb) was prepared as described previously [4] or developed by MBL (Nagoya, Japan) using a synthetic peptide corresponding to amino acids 750–765 of human FAM5C as the epitope. Mouse anti-von Willebrand factor (vWF) monoclonal antibody (mAb) (clone F8/86; Dako, Glostrup, Denmark), rabbit anti-mitochondrial Hsp70 pAb (#ab2799), mouse anti-calnexin mAb (AF18, #ab31290; Abcam, Cambridge, UK), mouse anti-GM130 mAb (clone 35/GM130, #610822), mouse anti-EEA1 mAb (clone 14/EEA1, #610457; BD Biosciences, San Jose, CA), mouse anti-ICAM-1 mAb (clone 15.2, #sc-107), mouse anti-VCAM-1 mAb (clone E-10, #sc-13160), rabbit anti-E-selectin pAb (#sc-14011; Santa Cruz Biotechnology Inc., Santa Cruz, CA), HRP-conjugated secondary Abs (GE Healthcare Bioscience, Pittsburgh, PA), fluorophore (Alexa 488 or 555)-conjugated secondary Abs (Molecular Probes, Eugene, OR), and fluorophore (FITC and Cy3)-conjugated secondary Abs (Jackson ImmunoResearch Laboratories, West Grove, PA) were purchased. 4′,6-Diamidino-2-phenylindole dihydrochloride (DAPI) was purchased from Nacalai Tesque, Inc. (Kyoto, Japan). FLAG-tagged FAM5C (FLAG–FAM5C) cDNA was prepared as described previously [4]. Human TNF-α (R&D Systems, Minneapolis, MN), IL-6, IL-1β, lipopolysaccharide (LPS) (Wako Pure Chemical Industries Ltd., Osaka, Japan), SP600215, SB203580 (Merck Millipore, Billerica, MA), BAY 11-7085, N-acetylcysteine (NAC) and thenoyltrifluoroacetone (TTFA; Sigma-Aldrich, St. Louis, MO) were purchased from commercial sources.

### Cell Culture and Transfection Experiments

Human aortic endothelial cells (HAECs), human coronary artery endothelial cells (HCAECs), human umbilical vein endothelial cells (HUVECs), and human microvascular endothelial cells (HMVECs) were purchased from Lonza (Basel, Switzerland) and cultured at 37°C using the EGM-2 BulletKit (Lonza). Human aortic SMCs (HASMCs) were purchased from Lonza and cultured at 37°C using the SmGM-2 BulletKit (Lonza). For the siRNA experiments, cells were transfected with Stealth RNAis for FAM5C or a nonsilencing negative control (Invitrogen, Carlsbad, CA) using Lipofectamine RNAiMAX (Invitrogen), according to the manufacturer's instructions. Electroporation-based transfection using Nucleofector (Lonza) was used for the transfection of FLAG–FAM5C cDNA. The cells were used for each experiment 48 h after transfection.

### RNA Extraction from Cultured Cells

Total RNAs were extracted from cultured cells using ReliaPrep RNA Cell Miniprep System (Promega, Madison, WI).

### Reverse Transcription PCR (RT-PCR)

Conventional RT–PCR assays were performed as described previously [10]. The primer sequences are presented in **Table 1**.

**Table 1:** Primer sequences and amplicon sizes.

Gene	Primer sequence	Size (bp)
***human FAM5A***	forward 5′-GAGCATCCGCCTGCTTGGCA-3′	506
	reverse 5′-CCTCGGCACATTGGCAGCGA-3	
***human FAM5B***	forward 5′-GGGGGCCATCAAGGTCACCG-3′	734
	reverse 5′-GCCCTTCGCCCAAGGCACAT-3′	
***human FAM5C***	forward 5′-CAACTGGAGAACAGCATGAAA-3′	539
	reverse 5′-TGAGGAACATCCGCTTACGC-3′	
***human GAPDH***	forward 5′-CTGATGCCCCCATGTTCGTC-3′	508
	reverse 5′-CACCCTGTTGCTGTAGCCAAATTC-3′	

### Immunofluorescence Microscopy

Immunofluorescence microscopic analyses of acetone-fixed HUVEC samples and formalin-fixed human coronary artery sections were performed as described previously [11], [12]. In brief, the samples were incubated with 1% bovine serum albumin in phosphate-buffered saline (PBS), then incubated with Blocking One (Nacalai Tesque) in PBS. The samples were stained with the indicated Abs and then with appropriate fluorophore-conjugated secondary Abs. The fluorescent signals were visualized with a confocal laser scanning microscope (LSM700 or LSM510 META, Carl Zeiss, Oberkochen, Germany).

### Histological Analysis

In this study, we used human coronary arteries that were obtained from 4 autopsy cases (4 males, 20–75 years) within 6 hours postmortem. Autopsies were performed at the University of Miyazaki Hospital in 2005 after written informed consent was provided by the families. Coronary arteries (proximal left anterior descending branch; Segment 6) were isolated from formalin-fixed heart as described [13]. Isolated arteries were cut transversely at 3 mm intervals and embedded in paraffin, and tissue sections were stained with hematoxylin-eosin (H-E) for morphological studies. These specimens were histologically categorized according to the American Heart Association classification [14], [15]. The lesions of adaptive intimal thickening (type I, early lesion), pathological intimal thickening/preatheroma (type III, intermediate lesion) and fibroatheroma/atheromatous plaque (Type IV, advanced lesion) were immunohistochemically examined in this study. The specimens were subjected to immunohistochemistry with the appropriate Abs. Signals were visualized with diaminobenzidine (Dako, Glostrup, Denmark). This study was approved by The Ethical Committee of the University of Miyazaki (Permit Number: 942) and performed in accordance with the ethical standards of the Declaration of Helsinki.

### RNA Extraction from Mice

Male C57BL/6J mice (8-week old) were intraperitoneally injected with murine TNF-α dissolved in 0.1% BSA in distilled water (500 µl of 1 ng/ml per mouse; Wako Pure Chemical Industries) (5 mice) or the equal volume of vehicle (5 mice) as a control, allowed to feed ad libitum, and 16 h later mice were anesthetized using isoflurane inhalation. After perfusion with physiological saline from the heart, the brain and aorta were dissected out and immediately total RNAs were extracted using ReliaPrep RNA Tissue Miniprep System (Promega). The animal experiments were approved by the Institutional Animal Care and Use Committee on the Ethics of Animal Experiments of Kobe University Graduate School of Medicine (Permit Number: P110711) and carried out according to the Kobe University Animal Experimental Regulations. All efforts were made to minimize suffering.

### Quantitative Real-time Reverse Transcription Polymerase Chain Reaction (qPCR)

The qPCR assays were performed using A ReverTra Ace qPCR RT Kit (Toyobo, Osaka, Japan) as described previously [11], [12]. Primers specific for mouse and human *FAM5C*, *ICAM1*, *VCAM1*, *SELE* (E-selectin), and *GAPDH* were purchased from Takara Bio (Otsu, Japan) or Life Technologies Corporation. *GAPDH* was used for normalization and the comparative threshold method was used to assess the relative abundance of the targets.

### Monocyte Adhesion Assay

HUVECs plated in 96-well plates at a density of 2.5×10^4^ cell/well and transfected with control or FAM5C siRNAs were incubated with TNF-α. Alternatively, HUVECs transfected with FLAG–FAM5C or empty vector (control) were plated at a density of 2.5×10^4^ cell/well and cultured in 96-well plates for 48 h. The monocyte/macrophage cell line THP-1 (5.0×10^4^ cell/well) was added to the monolayers of HUVECs, then incubated in RPMI-1640 media (Nacalai Tesque) supplemented with 10% fetal bovine serum at 37°C for 40 min. After the cells were washed three times with PBS, the numbers of THP-1 cells that adhered to the HUVEC monolayers were counted using microscopy and the values for three wells were averaged.

### Dihydroethidium Staining

HUVECs were incubated with 10 µM dihydroethidium (Molecular Probes) for 30 min in a dark chamber. After three washes with HEPES buffer (10 mM HEPES, 10 mM glucose, 140 mM NaCl, 5 mM KCl, 2 mM CaCl_2_, 5 mM NaHCO_3_, 0.6 mM Na_2_HPO_4_, 1.2 mM Na_2_SO_4_, pH 7.4), images were acquired using a fluorescence microscope (BZ-8100, Keyence, Osaka, Japan).

### Luciferase Reporter Assay

HUVECs (7.5×10^5^ cells) were cotransfected with 2 µg of pGL3–3kBpro, 0.5 µg of pTKβ-Gal and either the control or FAM5C siRNAs by an electroporation method with Amaxa Nucleofector Kits for HUVEC (Lonza), and then were plated in a well of 6-well plates. At 6 hours after transfection, the cells were washed and the medium was changed. The cells were cultured for the following 48 hours, and then incubated with 10 ng/ml TNF-α for the last 6 hours. The cells were lysed with 100 µl of reporter lysis buffer, and the luciferase activities were determined with Pikka Gene (Toyo Ink, Japan) and a luminometer (BioOrbit 1253 or Luminoskan Ascent). β-Galactosidase activities were measured using β-Galactosidase Enzyme Assay System with Reporter Lysis Buffer (Promega) according to the manufacturer's instructions. The luciferase activity was normalized by the β-galactosidase activity.

### Immunoblotting

Immunoblotting was performed as described previously [11], [12]. In brief, cells were washed twice with ice-cold PBS and lysed with buffer (20 mM Tris-HCl at pH 7.5, 150 mM NaCl, 1 mM Na_2_EDTA, 1 mM EGTA, 1% Triton X-100, 2.5 mM sodium pyrophosphate, 1 mM β-glycerophosphate, 1 µg/ml leupeptin, and 1 mM PMSF). The cell lysates were centrifuged at 12,000 *g* at 4°C for 10 min. The supernatant was mixed with 5× Laemmli buffer and boiled. The samples were subjected to SDS-PAGE and transferred to PVDF membranes. The membranes were blocked in 5% non-fat dry milk, and then incubated with the primary Abs, followed by incubation with HRP-conjugated secondary Abs. The signals were visualized by incubation with SuperSignal West Pico Chemiluminescent Substrate (Pierce), and then detected using the LAS-4000 mini imaging system (Fuji Film).

### Statistical Analysis

All experiments were performed at least three times, and the results are expressed as means ± standard errors of the means (SEM). Differences between groups were analyzed with one-way ANOVA, followed by the Tukey–Kramer post hoc test (PRISM5; GraphPad Software Inc., La Jolla, CA).

## Results

### FAM5C is Expressed and Localizes in the Golgi of Cultured Endothelial Cells

**Figure 1:**
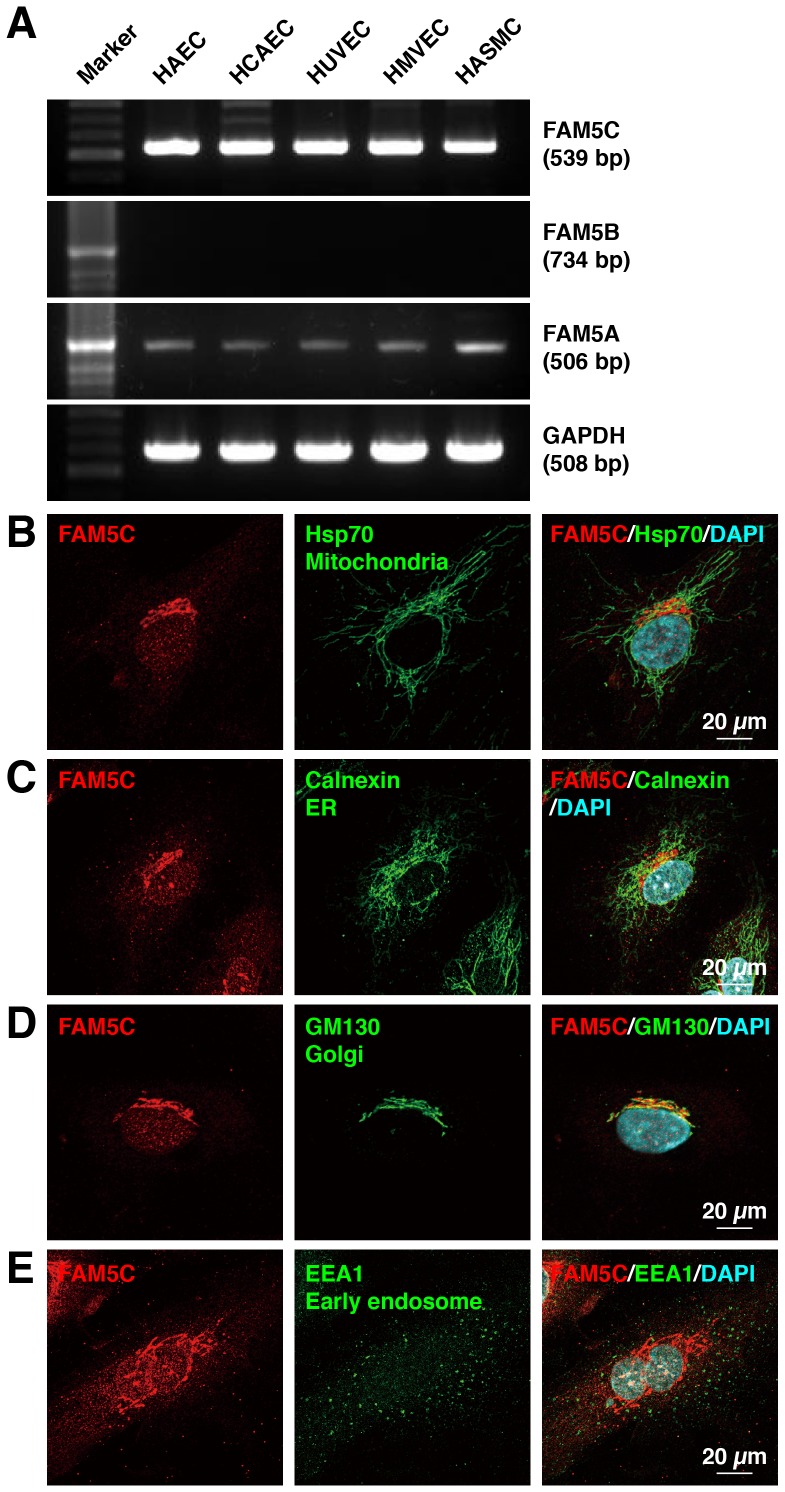
Expression and intracellular localization of FAM5C in cultured human endothelial cells. **A**, Expression of FAM5C mRNA. The expression of FAM5A, FAM5B, FAM5C, and GAPDH mRNAs was determined with conventional RT–PCR. **B–E**, Intracellular localization of FAM5C. HUVECs were stained with the indicated antibodies. The nuclei were stained with DAPI. The results shown are representative of three to four independent experiments, with identical results obtained. ER, endoplasmic reticulum.

We first examined whether FAM5C is expressed in endothelial cells. Human aortic SMCs were used as a control [9]. FAM5C mRNA was detected in various cultured human endothelial cells and aortic SMCs (**Fig. 1A**). FAM5A mRNA was also detected in these cells, whereas FAM5B mRNA was not (**Fig. 1A**). Intracellular localization of FAM5C was analyzed by immunofluorescence microscopy. In HUVECs, the immunofluorescence signal for FAM5C was mainly observed in the perinuclear region (**Fig. 1** **, B–E**). In the perinuclear region, the immunofluorescence signal for FAM5C colocalized with that of the Golgi marker GM130, but not with those for mitochondrial protein Hsp70, the endoplasmic reticulum protein calnexin, or early endosome antigen 1 (EEA1). These findings indicate that FAM5C is expressed and localizes in the Golgi of cultured endothelial cells.

### FAM5C Is Expressed in Endothelial Cells *in Vivo*

**Figure 2:**
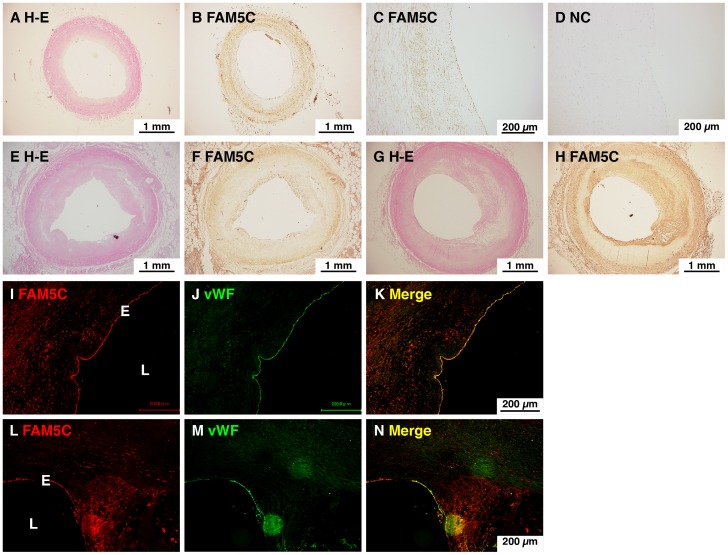
Expression of FAM5C in the endothelium of human coronary aretery. **A–H**, Immunohistochemistry of human coronary arteries. Specimens of coronary artery from a 20-year-old male without coronary artery disease (**A–D**), a 50-year-old man with old myocardial infarction (**E, F**) and a 75-year-old man with hepatocellular carcinoma (**G, H**) were subjected to immunohistochemistry. Hematoxylin-eosin (**H–E; A, E, G**); FAM5C (**B, C, F, H**); IgG (**D**). **I–N**, Double immunofluorescence staining for FAM5C and vWF in human coronary artery. Specimens of coronary arteries obtained from a 50-year-old man with old myocardial infarction (**I–K**) and a 68-year-old man with chronic renal failure (**L–N**) were subjected to double immunofluorescence staining for FAM5C and vWF. FAM5C (**I, L**); vWF (**J, M**); merge (**K, N**). E, endothelium; L, lumen.

We then analyzed *in vivo* expression of FAM5C in human coronary arteries by immunohistochemistry and immunofluorescence microscopy. In human coronary artery without apparent atherosclerotic plaques, FAM5C immunostaining was diffusely observed within the vessel wall (**Fig. 2** **, A–D**). In human coronary artery with different degrees of atherosclerotic plaques, FAM5C immunostaining was diffusely observed within the vessel wall and, of note, relatively strong signals were observed in the luminal side of the thickened neointima and the media (**Fig. 2** **, E–H**). High magnification views of double immunofluorescence staining for FAM5C and the endothelial marker vWF showed that a relatively strong signal for FAM5C was observed in the endothelium that was positive for vWF staining and a weak FAM5C signal was observed in the neointima (**Fig. 2** **, I–N**). These findings show that FAM5C is expressed in endothelial cells of human coronary arteries.

### Colocalization of FAM5C with ICAM-1 and VCAM-1 in the Endothelium of Human Coronary Arteries

**Figure 3:**
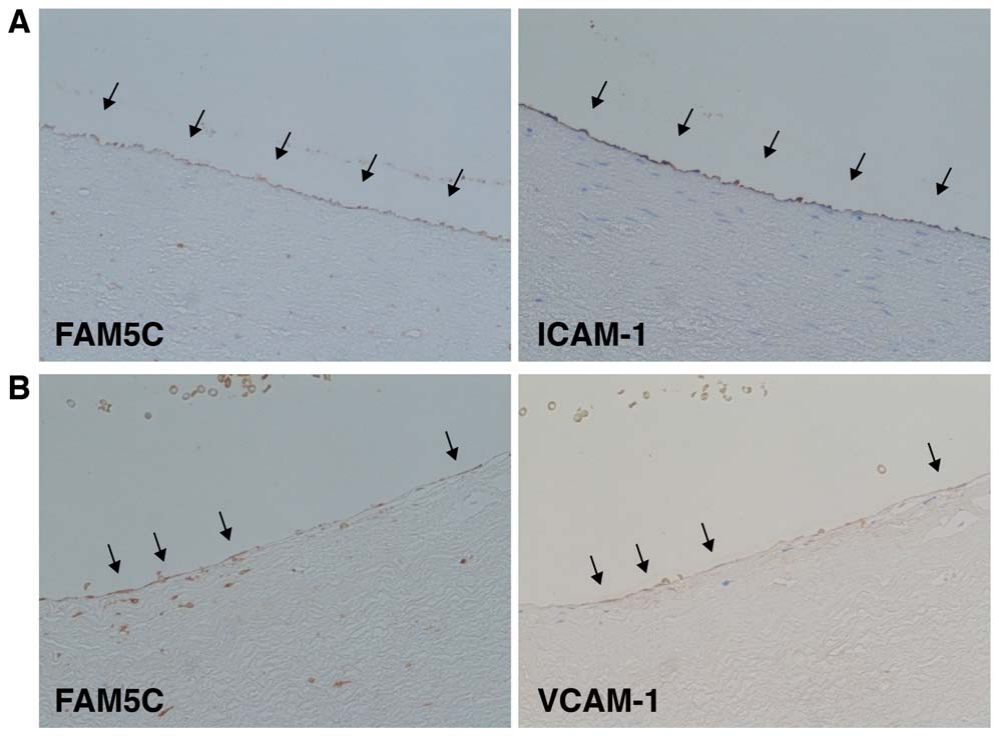
Colocalization of FAM5C with ICAM-1 and VCAM-1 in the endothelium of human coronary arteries. Serial sections of coronary arteries obtained from a 68-year-old man with chronic renal failure (**A**) and a 75-year-old man with hepatocellular carcinoma (**B**) were subjected to immunofluorescence staining for FAM5C and ICAM-1 (**A**) or VCAM-1 (**B**).

To investigate whether there is a link between FAM5C and vascular inflammation, we performed the immunohistochemistry of leukocyte adhesion molecules using human coronary artery sections. FAM5C staining was observed in the endothelium in which staining of ICAM-1 (**Fig. 3A**) and VCAM-1 (**Fig. 3B**) was observed. These results show a possible link between FAM5C and vascular inflammation.

### Overexpression of FAM5C Increases Expression of Leukocyte Adhesion Molecule mRNAs

**Figure 4:**
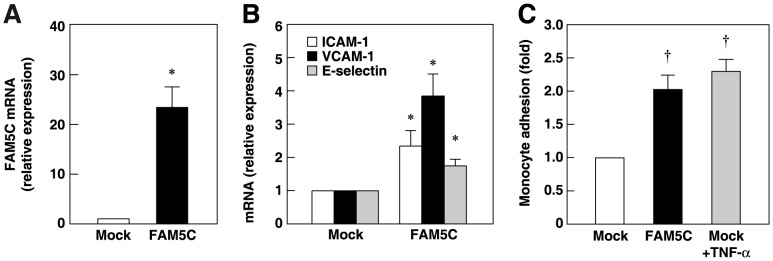
Upregulation of leukocyte adhesion molecules by FAM5C. **A and B**, Total RNA was extracted from HUVECs 48 h after transfection with 0.1 µg of FLAG (Mock) or FLAG–FAM5C (FAM5C) cDNAs. An increase in FAM5C mRNA levels (**A**) (n = 3). and upregulation of ICAM-1, VCAM-1 and E-selectin mRNA levels (**B**) (n = 3) were analyzed by qPCR. **C**, 48 h after transfection, monocyte adhesion assays were performed and the numbers of monocytes that adhered to the HUVEC monolayers were analyzed (n = 5). HUVECs incubated with 10 ng/ml TNF-α for the last 16 h were used as the controls. *P<0.05, ^†^P<0.01 vs Mock.

We then analyzed the effect of the overexpression of FAM5C on the expression of the leukocyte adhesion molecules. Transfection of FLAG–FAM5C cDNA increased FAM5C mRNA levels in HUVECs compared with those in mock-transfected cells (**Fig. 4A**). Transfection of FLAG–FAM5C cDNA increased the mRNA levels of ICAM-1, VCAM-1 and E-selectin (**Fig. 4B**). Consistent with these findings, the transfection of FLAG–FAM5C cDNA increased the numbers of monocytes that adhered to HUVEC monolayers, which were similar to those that adhered to TNF-α-stimulated HUVECs (**Fig. 4C**). Therefore, the overexpression of FAM5C increased the endothelial expression of mRNAs for adhesion molecules, leading to enhanced monocyte adhesion.

### Overexpression of FAM5C Increases the Production of Reactive Oxygen Species (ROS) and Nuclear Factor-κB (NF-κB) Activity in Cultured Endothelial Cells

**Figure 5:**
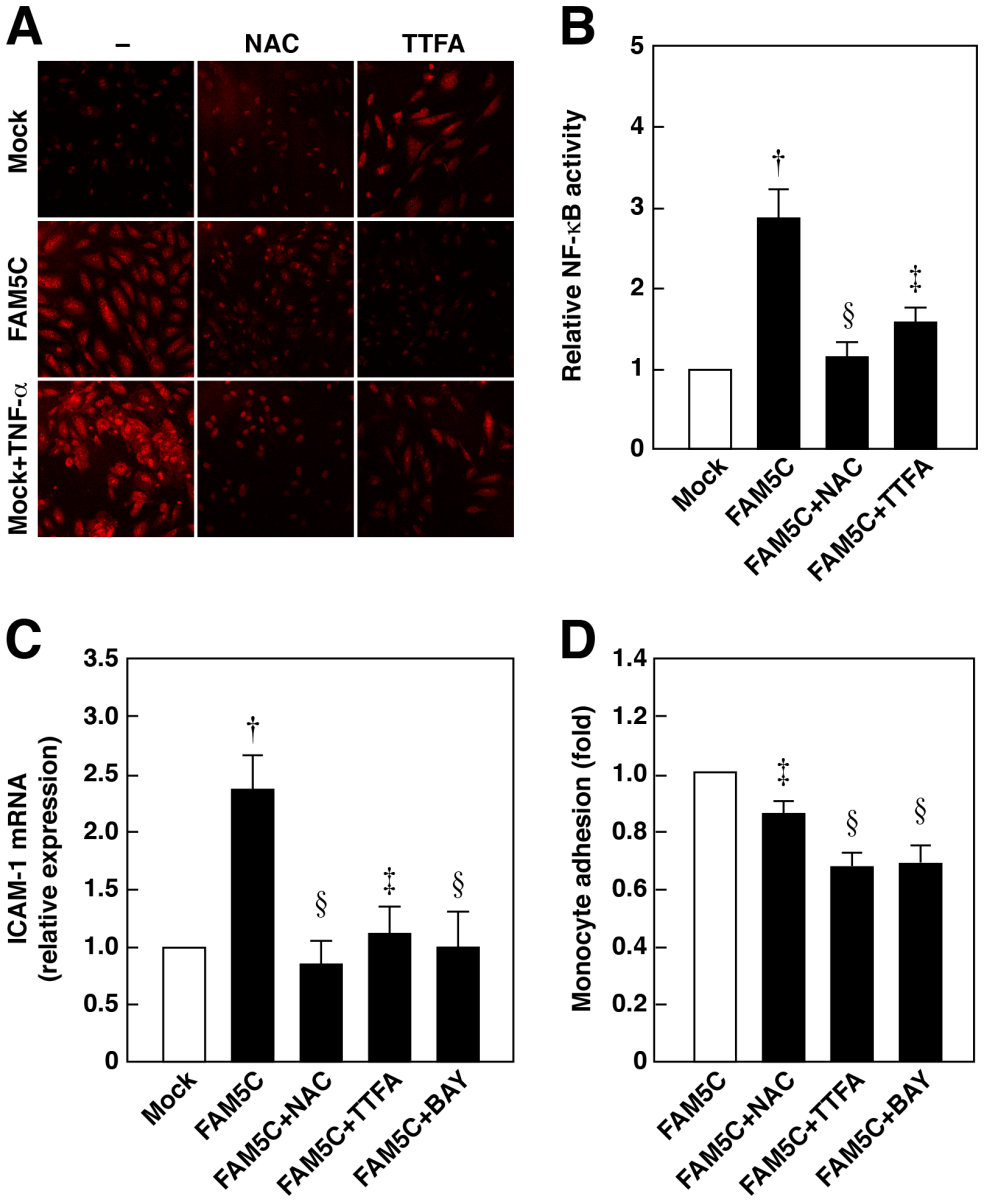
FAM5C increases ROS production and NF-κB activity. **A and B**, HUVECs were transfected with 0.1 µg of FLAG (Mock) or FLAG–FAM5C (FAM5C) cDNAs and then incubated with 10 mM NAC or 10 µM TTFA for 16 h. ROS production assessed by dihydroethidium staining (**A**) and NF-κB activity (**B**) (n = 3–5) were analysed. **C and D**, HUVECs were transfected with 0.1 µg of FLAG (Mock) or FLAG–FAM5C (FAM5C) cDNAs and then incubated with 10 mM NAC, 10 µM TTFA, or 10 µM BAY 11-7085 (BAY) for 16 h. Involvement of ROS and NF-κB in the increase in FAM5C-induced ICAM-1 mRNA expression (**C**) (n = 4–5) and monocyte adhesion to HUVECs (**D**) (n = 6) were analyzed. ^†^P<0.01 vs Mock, ^‡^P<0.05, ^§^P<0.01 vs FAM5C.

We examined whether the overexpression of FAM5C increases ROS production and NF-κB activity. TNF-α was used as the positive control to increase ROS production in HUVECs. This increase was blocked by cotreatment with NAC, a powerful antioxidant, or TTFA, an inhibitor of the mitochondrial electron transport chain complex II, although TTFA itself slightly increased ROS production (**Fig. 5A**). The increase in ROS production by TTFA is consistent with previous papers [16], [17]. The overexpression of FAM5C increased ROS production in HUVECs, which was blocked by cotreatment with NAC or TTFA (**Fig. 5A**). The transfection of FLAG–FAM5C cDNA increased the luciferase activity, indicating an increase in NF-κB activity (**Fig. 5B**). The FAM5C-induced increase in NF-κB activity was blocked by cotreatment with NAC or TTFA (**Fig. 5B**). Consistent with these findings, the FAM5C-induced increase in ICAM-1 expression was blocked by cotreatment with NAC, TTFA, or the NF-κB inhibitor BAY 11-7085 (**Fig. 5C**). Similarly, the numbers of monocytes that adhered to FLAG–FAM5C-transfected HUVEC monolayers were decreased by cotreatment with NAC, TTFA, or BAY 11-7085 (**Fig. 5D**). Therefore, the overexpression of FAM5C increased ROS production and NF-κB activity, leading to the upregulated expression of ICAM-1 and enhanced monocyte adhesion.

### FAM5C is Upregulated in Response to Inflammatory Stimuli

**Figure 6:**
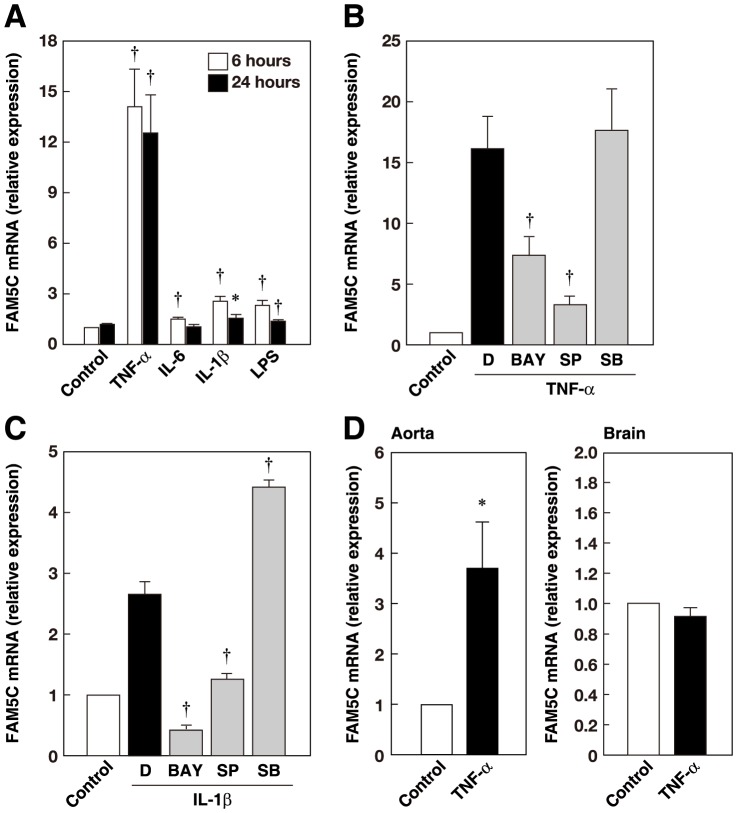
Upregulation of FAM5C mRNA by inflammatory stimuli in an NF-κB- and JNK-dependent manner. **A**, HUVECs were cultured in the presence of 10 ng/ml TNF-α, 50 ng/ml IL-6, 10 ng/ml IL-1β, or 10 ng/ml LPS for 6 or 24 h (n = 11). **B and C**, HUVECs were incubated with 10 µM BAY 11-7085 (BAY), SP600125 (SP), SB203580 (SB), or DMSO (D) for 30 min and then cultured in the presence of 10 ng/ml TNF-α (**B**) (n = 8) or 10 ng/ml IL-1β (**C**) (n = 5) for 6 h. Values are expressed as the fold increase over the unstimulated control. ^*^P<0.05, ^†^P<0.01 vs control (**A**); *P<0.05, ^†^P<0.01 vs DMSO (**B, C**). **D**, Upregulation of FAM5C mRNA expression in aortas of TNF-α-injected mice. RNA was extracted from mouse aortas and brains of the control (n = 5) and TNF-α-injected (n = 5) mice.

To investigate how expression of FAM5C is regulated in endothelial cells, we analyzed the effects of inflammatory stimuli on FAM5C mRNA expression. Incubation with TNF-α, IL-6, IL-1β, or LPS significantly increased FAM5C mRNA levels in HUVECs (**Fig. 6A**). Treatment of these cells with the NF-κB inhibitor BAY 11-7085 or the JNK inhibitor SP600215, but not the p38 inhibitor SB203580, inhibited the increase in the FAM5C mRNA expression induced by TNF-α (**Fig. 6B**) and IL-1β (**Fig. 6C**). Therefore, FAM5C is upregulated in response to inflammatory stimuli, and TNF-α and IL-1β increase FAM5C mRNA expression in an NF-κB- and JNK-dependent manner. We then examined whether TNF-α could increase FAM5C expression *in vivo*. Consistent with the results in HUVECs, the FAM5C mRNA levels were elevated in the aorta, but not in the brain, of mice following an intraperitoneal injection of TNF-α, when assessed with qPCR (**Fig. 6D**).

### Involvement of FAM5C in the Increase in the TNF-α-induced Expression of Leukocyte Adhesion Molecule mRNAs

**Figure 7:**
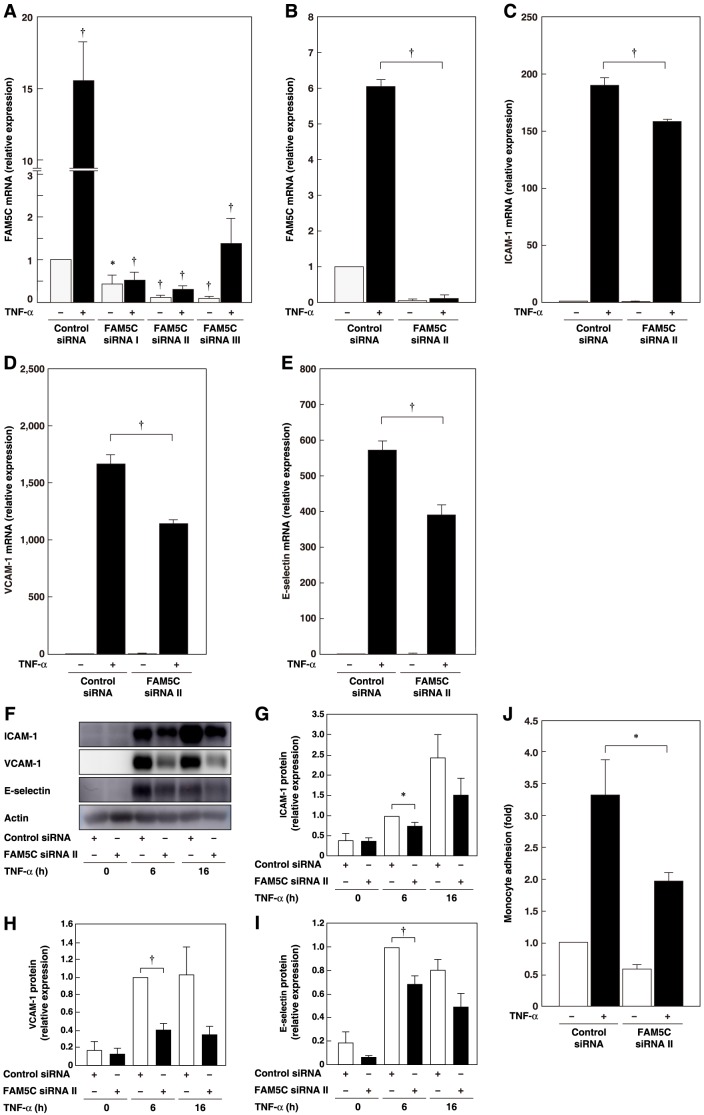
Involvement of FAM5C in the TNF-α-induced upregulation of leukocyte adhesion molecules. **A**, Knockdown of FAM5C. HUVECs transfected with control or FAM5C siRNAs were cultured for 16 h, and were then cultured in the presence of 10 ng/ml TNF-α for 6 h (n = 6). *P<0.05, ^†^P<0.01 vs control. **B–E**, Inhibition of the increase in the TNF-α-induced expression of FAM5C, ICAM-1, VCAM-1 and E-selectin mRNAs by FAM5C knockdown. HUVECs transfected with control or FAM5C siRNAs were cultured for 48 h, and were then cultured in the presence of 10 ng/ml TNF-α for 6 h. FAM5C (**B**), ICAM-1 (**C**), VCAM-1 (**D**), and E-selectin (**E**) mRNA levels were analyzed (n = 4). ^†^P<0.01 vs control siRNA. **F–I**, Inhibition of the increase in the TNF-α-induced expression of ICAM-1, VCAM-1 and E-selectin proteins by knockdown of FAM5C. HUVECs transfected with control or FAM5C siRNAs were cultured for 48 h, and were then cultured in the presence of 10 ng/ml TNF-α for the indicated times. Representative blots (**F**) and densitometric analysis of band intensities of ICAM-1 (**G**), VCAM-1 (**H**) and E-selectin (**I**) were shown (n = 4). *P<0.05, ^†^P<0.01 vs control siRNA. **J**, Inhibition of the increase in TNF-α-induced monocyte adhesion to HUVECs by knockdown of FAM5C. HUVECs transfected with control or FAM5C siRNAs were cultured for 48 h, and were then cultured in the presence of 10 ng/ml TNF-α for 16 h (n = 4–6). *P<0.05, ^†^P<0.01 vs control.

To analyze the role of FAM5C in the TNF-α-induced expression of leukocyte adhesion molecules, we knocked down by using siRNAs. HUVECs were transfected with siRNAs and 16 h later, the cells were further incubated with TNF-α for 6 h. Three different FAM5C siRNAs significantly reduced the FAM5C mRNA levels under untreated and TNF-α-stimulated conditions (**Fig. 7A**). Next, HUVECs were transfected with siRNA and 48 h later, the cells were incubated again with TNF-α for 6 h. In this experiment, FAM5C siRNA significantly reduced FAM5C mRNA expression under untreated and TNF-α-stimulated conditions (**Fig. 7B**). Under this experimental condition, TNF-α markedly increased the mRNA levels of ICAM-1, VCAM-1 and E-selectin, and these increases were significantly inhibited by FAM5C siRNA (**Fig. 7C–E**). Consistent with these findings, TNF-α increased the protein levels of ICAM-1, VCAM-1 and E-selectin, and these increases were inhibited by FAM5C siRNA (**Fig. 7** **, F–I**). Furthermore, TNF-α increased the number of monocytes that adhered to the HUVEC monolayers and this increase was significantly inhibited by FAM5C siRNA (**Fig. 7J**). These findings indicate the involvement of FAM5C in increasing the TNF-α-induced expression of adhesion molecule mRNAs, causing monocytes to adhere to endothelial cells.

### Involvement of FAM5C in the Increase in the TNF-α-induced Production of ROS and NF-κB Activity in Cultured Endothelial Cells

**Figure 8:**
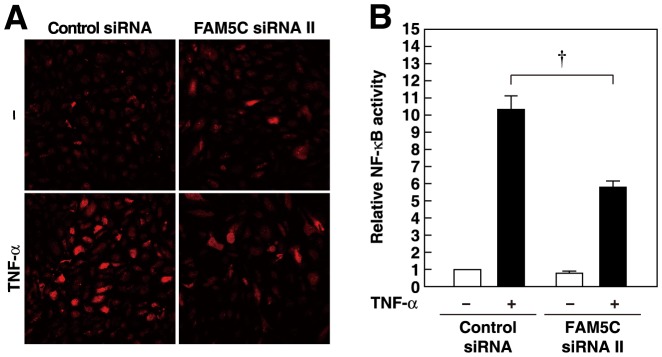
Involvement of FAM5C in the increases in ROS production and NF-κB activity. HUVECs transfected with control or FAM5C siRNAs were cultured for 48 h, and were then cultured in the presence of 10 ng/ml TNF-α for 6 h. The increase in TNF-α-induced ROS production (**A**) and NF-κB activity (**B**) by knockdown of FAM5C. The results shown are representative of three independent experiments (**A**). The luciferase activity was normalized by the β-galactosidase activity (**B**) (n = 4).

We examined the effect of FAM5C on the production of ROS. TNF-α increased the production of ROS in HUVECs, and this increase was inhibited by FAM5C siRNA (**Fig. 8A**). We then analyzed NF-κB activity using luciferase assays [11]. TNF-α increased NF-κB activity in HUVECs, and this increase was significantly inhibited by FAM5C siRNA (**Fig. 8B**). These results indicate that FAM5C is involved in the TNF-α-induced ROS production and NF-κB activity.

## Discussion

Our results suggest that inflammatory stimuli, such as TNF-α, induce the upregulated expression of FAM5C though the NF-κB and JNK signaling pathways, and that FAM5C increases the mRNAs of leukocyte adhesion molecules, such as ICAM-1, VCAM-1 and E-selectin, through the ROS–NF-κB signaling pathway, thus causing enhanced monocyte adhesion to endothelial cells.

Several research groups have reported a genetic association between *FAM5C* and the onset or severity of diseases such as cancers [5], [7], periodontitis [8], [18] and IHD [9], [19]. The results of most studies implicate FAM5C as a disease marker, although the association between *FAM5C* and periodontitis is controversial [8], [18]. However, the contribution of FAM5C to the phenotype of each disease remains largely unknown. Furthermore, two studies that examined FAM5C mRNA expression levels in a specific disease state found that FAM5C mRNA levels are reduced in tongue squamous cell carcinoma [5] and *FAM5C* is hypermethylated in gastric cancer [7]. FAM5C mRNA is expressed in the human aorta and its transcription levels are reduced with increasing passages of cultured SMCs, suggesting that FAM5C plays a role in the proliferation and senescence of this cell type [9]. Consistent with this idea, our results of immunohistochemistry showed relatively strong FAM5C staining in the thickened neointima, where proliferation of SMCs occurs. However, the mechanism regulating *FAM5C* gene expression is unknown. Although FAM5C is homologous to BMP- and RA-inducible FAM5A, it is not induced by BMP or RA in cultured neuronal cells [3]. Our results show that FAM5C mRNA levels are increased by inflammatory stimuli, such as TNF-α, IL-6, IL-1β and LPS, in cultured endothelial cells. It is well known that the NF-κB and JNK signaling pathways contribute to inflammation and endothelial cell activation. The TNF-α- and IL-1β-induced increases in FAM5C expression were inhibited by NF-κB and JNK inhibitors. Although we did not examine the effects of these inhibitors on the increase in FAM5C expression induced by IL-6 and LPS, the NF-κB and JNK signaling pathways might be involved in these increases because these stimuli activate the NF-κB and JNK pathways.

The function of FAM5C is poorly understood. The forced expression of FAM5C reduced cell-cycle progression in NIH3T3 cells [3], but increased the proliferation, migration and invasion of pituitary gonadotrope cells [4]. The latter phenotype is probably relevant to the phenotypic changes in vascular SMCs that are associated with the formation and vulnerability of atherosclerotic plaques, which has implications for acute coronary syndrome, including myocardial infarction [9]. In contrast to these findings, overexpression of FAM5C did not enhance the proliferation of endothelial cells (data not shown). However, our results suggest that the TNF-α-induced upregulation of leukocyte adhesion molecule mRNAs, such as ICAM-1, VCAM-1 and E-selectin, and TNF-α-enhanced monocyte adhesion to endothelial cells were partly inhibited by the knockdown of FAM5C. Furthermore, the forced expression of FAM5C upregulated the expression of leukocyte adhesion molecule mRNAs and enhanced monocyte adhesion to endothelial cells. Although the increases in the leukocyte adhesion molecule mRNAs caused by the forced expression of FAM5C were much smaller than those caused by TNF-α, the monocyte adhesion to endothelial cells was similar under these two conditions, suggesting that the forced expression of FAM5C may have other effects on monocyte adhesion besides the upregulation of leukocyte adhesion molecule mRNAs. Although several reports have provided evidence of a possible link between FAM5C and inflammation, the biological functions relevant to inflammation are poorly identified. Our findings that the expression of FAM5C is upregulated by inflammatory stimuli and that FAM5C contributes to leukocyte adhesion implicate FAM5C as a mediator of vascular inflammation. Because vascular inflammation is important in acute coronary syndrome, the association between FAM5C and myocardial infarction may involve the effects of FAM5C on endothelial cell activation. Therefore, the results of the present study show for the first time the regulation of FAM5C expression and its important function in endothelial cells under inflammatory conditions. However, our pathological study has several limitations. The sample size was small, and the coronary artery samples were taken from patients in a wide age range and showed different degrees of atherosclerosis.

Our results also suggest that the overexpression of FAM5C increases ROS production in endothelial cells. This increased ROS production contributes to NF-κB activation because NF-κB activation was inhibited by NAC and TTFA, both of which reduce ROS production. Because the TNF-α-induced NF-κB activation contributes to FAM5C expression, it might be possible that there is a positive feedback mechanism where FAM5C-induced NF-κB activation induces FAM5C expression. ROS may originate from various sources, but it has been demonstrated that superoxide production occurs in mitochondria isolated from bovine aortic endothelial cells [20]. Our results suggest that the mitochondrial electron transport chain complex II inhibitor TTFA reduced the ROS production induced by overexpressed FAM5C in HUVECs. The inhibition of ROS production by TTFA suggests that the mitochondrial respiratory chain is a source of ROS. However, the mechanism by which FAM5C increases ROS production by the mitochondria is unknown. It has been reported that FAM5C localizes in the mitochondria in human gonadotropinoma cells [4]. However, our results suggest that FAM5C localized in the Golgi in HUVECs. According to the Universal Protein Resource Knowledgebase (UniProtKB, http://www.uniprot.org/uniprot), the FAM5C protein has a signal peptide at its N-terminus and is therefore presumably a secreted protein. Consistent with this, Tanaka et al. recently reported that FAM5C is a soluble osteoblast differentiation factor linking muscle to bone [6]. In that paper, the conditioned medium from FAM5C-overexpressing and -suppressed cells effectively increased and reduced the expression levels of osteoblast differentiation marker genes in osteoblastic cells, respectively. Therefore, although verification is needed, our results suggest that FAM5C secreted by endothelial cells might increase ROS production in an autocrine/paracrine fashion.

ROS are probably continuously produced in all living cells, including endothelial cells, through the reduction of molecular oxygen by the electron transport chain in the mitochondria, producing superoxide, which is subsequently dismutated to hydrogen peroxide [21]. However, in a diseased state, ROS overwhelm the antioxidant defenses, causing oxidative damage, often referred to as “oxidative stress”, possibly leading to the attenuation of the vasodilatory response mediated by endothelial nitric oxide in blood vessels. Therefore, the ROS production induced by the overexpression of FAM5C in the endothelium may increase the oxidative stress in the vascular wall, which is relevant to atherosclerosis.

SNPs of *FAM5C* correlate with the incidence of myocardial infarction and the expression levels of FAM5C in aortic SMCs [9]. Increased FAM5C expression in aortic SMCs is suggested to be a risk factor for myocardial infarction. Because ROS stimulate the proliferation and migration of vascular SMCs [22], and because they contribute to plaque instability [23], an increase in FAM5C expression in the coronary artery may be associated with the severity of coronary artery atherosclerosis and plaque vulnerability. No relationship between *FAM5C* SNPs and inflammatory biomarkers [19] has been reported. However, because FAM5C is presumably a secreted protein, it will be interesting to investigate whether FAM5C in the blood is a biomarker of vascular inflammation.
